# Effects of different therapeutic methods on the 90-day prognosis of patients with HBV-ACLF: A systematic review and network meta-analysis

**DOI:** 10.1097/MD.0000000000046388

**Published:** 2025-12-19

**Authors:** Yin Hua, Huaqiang Liu, Yuqin He, Shousheng Liu, Yongning Xin

**Affiliations:** a Department of Infectious Disease, Qingdao Clinical Medical College Affiliated to Nanjing Medical University, Qingdao Municipal Hospital, Qingdao, China; b Department of Pharmacy, Qingdao Sixth People’s Hospital, Qingdao, China; c Department of Gastroenterology, Wuxi Clinical Medical College Affiliated to Nanjing Medical University, Wuxi, China; d Clinical Research Center, Qingdao Municipal Hospital, University of Health and Rehabilitation Sciences, Qingdao, China; e Department of Infectious Disease, Qingdao Municipal Hospital, University of Health and Rehabilitation Sciences, Qingdao, China.

**Keywords:** granulocyte colony-stimulating factors, HBV-ACLF, mesenchymal stem cells, meta-analysis, plasma exchange

## Abstract

**Background:**

The short-term mortality rate of hepatitis B virus related acute-on-chronic liver failure (HBV-ACLF) is relatively high, and the optimal therapeutic method for HBV-ACLF is still controversial. This study aimed to investigate the effects of different therapeutic methods on 90-day prognosis of HBV-ACLF patients.

**Methods:**

PubMed, Embase, and Cochrane Library were searched from their inception date up to February 2, 2023. RCTs or cohort studies related to the treatment of HBV-ACLF with different therapies were identified. Therapeutic methods focused on plasma exchange (PE), mesenchymal stem cell (MSC), granulocyte colony-stimulating factor (G-CSF), glucocorticoid (GC), double plasma molecular absorption system (DPMAS), and nucleos(t)ide analogues. The primary outcome was the 90-day survival rate. The results for binary variables were calculated using odds ratio (OR), with a corresponding 95% confidence interval (CI).

**Results:**

A total of 16 studies were included in this study, which contained the therapeutic methods of PE, GC, G-CSF, MSC, DPMAS + PE, MSC + PE. Compared to nucleos(t)ide analogues, treatment with MSC + PE, DPMAS + PE, G-CSF, MSC and PE increased the odds of 90-day survival rate (OR = 4.58, 95% CI: 1.95–10.75; OR = 2.95, 95% CI: 1.07–8.12; OR = 2.32, 95% CI: 1.15–4.69; OR = 2.36, 95% CI: 1.14–4.91; OR = 1.91, 95% CI: 1.42–2.77), respectively. The odds of the 90-day survival rate after being treated by MSC + PE were superior to PE (OR = 2.40, 95% CI: 1.05–5.51) and GC treatment (OR = 2.86, 95% CI: 1.01–8.09). MSC + PE exhibited the highest likelihood (0.92) of being the optimal therapeutic method in improving the prognosis of patients with HBV-ACLF. For single-drug regimens, G-CSF (0.58) ranks the highest.

**Conclusion:**

This meta-analysis showed that MSC + PE was the most effective therapy in improving the 90-day prognosis, and G-CSF was the potential optimal monotherapy in improving the 90-day prognosis of HBV-ACLF patients.

## 1. Introduction

Acute-on-chronic liver failure (ACLF) is defined as the appearance of jaundice and coagulopathy, regarded as the initial clinical manifestation of acute hepatic injury, complicated with ascites and/or encephalopathy within 4 weeks of onset, with previously diagnosed or undiagnosed chronic liver disease/cirrhosis. Abundant clinical evidence has proven that ACLF is associated with a high 28-day mortality.^[1]^ In Asia, hepatitis B virus (HBV) infection is the leading cause of ACLF, which is defined as hepatitis B virus related acute-on-chronic liver failure (HBV-ACLF), with a high overall mortality rate ranging from 30% to 70%.^[2]^

Multiple therapeutic methods have been developed to treat the HBV-ACLF and currently, 3 main treatment options are available for HBV-ACLF: medication, artificial liver support systems (ALSS), and liver transplantation (LT).^[1]^ Until now, LT remains the only truly effective therapy for HBV-ACLF, although it is limited by a scarcity of donors, extended waitlists, high costs, and numerous complications.^[3]^ Besides LT, ALSS is commonly used for the treatment of HBV-ACLF.^[4]^ One frequently-used ALSS is plasma exchange (PE), which can remove the metabolic toxins and small molecules from the bloodstream via a membrane plasma separator, and then replaces these removed substances with fresh frozen plasma.^[5]^ The short supply of fresh frozen plasma is the main disadvantage of using PE as the treatment method for HBV-ACLF.^[6]^ Furthermore, the DPMAS of PE is capable of adsorbing medium- and macro-molecular toxins, including inflammatory mediators and bilirubin, and could avoid the issue of blood source shortages associated with PE.^[7]^

Various medication treatment options are available for the management of HBV-ACLF, including glucocorticoid (GC), granulocyte colony-stimulating factor (G-CSF), mesenchymal stem cell (MSC), and nucleos(t)ide analogues (NAs).^[8–11]^ Almost all of these methods can alleviate the symptom of patients with HBV-ACLF by promoting liver cell regeneration and preventing further liver cell damage.^[1]^ GC is a kind of important immunosuppressive and anti-inflammatory drugs that have been used in immune- and alcoholic-related ACLF, but their effects on patients with HBV-ACLF remain controversial.^[8]^ Both G-CSF and MSC can improve the prognosis of HBV-ACLF patients by promoting liver cell regeneration through stem cells.^[9,10]^ G-CSF could facilitate the bone marrow-derived stem cells enter into the peripheral circulation, thereby improving the hepatocellular status of HBV-ACLF patients.^[10]^ Theoretically, stem cell therapy possesses great potential for patients with ACLF and patients exhibiting critically impaired liver function. MSC could exert immune-regulatory functions to alleviate the immunosuppressive state in the late stage of ACLF, thereby reducing the likelihood of infection occurrence.^[9]^ HBV reactivation is the leading cause of HBV-ACLF, and drug resistance to NAs or unauthorized withdrawal of NAs can lead to the HBV reactivation. Hence, effective antiviral drugs can prevent the onset of HBV-ACLF effectively.^[11]^

Accumulated studies have investigated the effects of different therapeutic methods on patients with HBV-ACLF, but which is the optimal therapy for HBV-ACLF in terms of the 90-day prognosis remains unclear. In clinical practice, physicians must discern from a broad spectrum of treatment options to determine the most secure, cost-effective, and efficient regimen tailored to the individual.^[12]^ Therefore, a systematic review and network meta-analysis was conducted to compare the effects of different therapeutic methods on the 90-day prognosis in patients with HBV-ACLF.

## 2. Materials and methods

### 2.1. Search strategy

The databases of Cochrane Library, Embase, and PubMed were searched from their inception date until February 2, 2023, along with ClinicalTrials.gov and the World Health Organization trial registers. Only articles that were published in English were selected, and a combination of subject terms and free-text words was used to search the electronic databases. Treatment options for HBV-ACLF included PE, GC, G-CSF, MSC, and DPMAS, were searched using the following search terms: PE (also searched as exchange plasma and plasma exchanges), GC (also searched as glucocorticoid and glucocorticoid effect), G-CSF (also searched as granulocyte colony-stimulating factor), MSC (also searched as stem cell and mesenchymal), and DPMAS (also searched as double plasma molecular absorption system). Additionally, relevant study reference lists were reviewed to identify additional studies. Data from unpublished clinical trials were deemed unreliable and, consequently, were excluded from this study.

### 2.2. Study selection

A network meta-analysis that involved screening studies based on specific criteria was conducted. The inclusion criteria were as follows: randomized controlled trials (RCTs), prospective or retrospective cohort studies in the study design; subjects in study were aged ≥ 18 years and diagnosed with HBV-ACLF based on APASL criteria; study interventions that included at least one of these methods: PE, DPMAS, GC, G-CSF, MSC, and antiviral medications, provision of energy and vitamin supplements, and the use of blood products); and the outcome encompass a 90-day survival rate, as well as liver function parameters such as alanine aminotransferase (ALT), total bilirubin (TBIL), and model of end-stage liver disease (MELD) score, along with the side effects of the treatment approach. The exclusion criteria including: co-infection with other hepatitis viruses or autoimmune diseases; history of alcohol abuse or hepatoxic drugs within the past 6 months; original literature materials and data were unavailable; and review articles, case reports, letters, editorials, nonhuman studies, and duplicate studies.

### 2.3. Data extraction and quality assessment

To ensure this study complies with the inclusion and exclusion criteria strictly, 2 researchers conducted a thorough screening of the literature, extracting and sorting data based on the study’s characteristics, patient characteristics, interventions, and outcome indicators, respectively. Literature that aligned with this research was chosen after reviewing the titles, abstracts, and full texts. Any discrepancies were resolved by discussion with a third reviewer. The following information of each included study was recorded: publication year, first author, research methodology, number of patients, age, gender, and diagnostic criteria, and details of interventions and outcome measures. The quality of RCT was assessed using Cochrane’s recommended bias risk assessment tool, with each item evaluated as high, low, or unclear risk. Cohort study quality was evaluated using the Newcastle-Ottawa scale. A study with a score of 6 or more was considered to be of high quality. Review Manager (version 5.3) was used by 2 independent researchers to evaluate the article quality and risk of bias, and any subsequent disagreements were resolved by a third reviewer.

### 2.4. Data synthesis and statistical analyses

The random-effects model was used to calculate the odds ratio (OR) and 95% CI for each outcome in the network meta-analysis. The threshold for statistical significance was established at a 2-tailed *P*-value of <.05, and all statistical computations were conducted utilizing STATA (version 15.0). A frequentist model was employed in this network meta-analysis to conduct all treatment comparisons through a network graph for each outcome. The loop consistency was evaluated utilizing node splitting to identify the similarities between direct and indirect treatment effects, with significant inconsistency considered present when the difference between the direct and indirect estimates yielded a *P*-value of <.05. The surface under the cumulative ranking curve (SUCRA) probabilities were used to appraise the treatment strategies for each outcome, where the high SUCRA probabilities signify an increased likelihood of the optimal treatment regimen in every simulation. Funnel plots were conducted using STATA (version 15.0) to assess the publication bias of each study.

## 3. Results

### 3.1. Characteristics of the included studies

**Figure 1.**
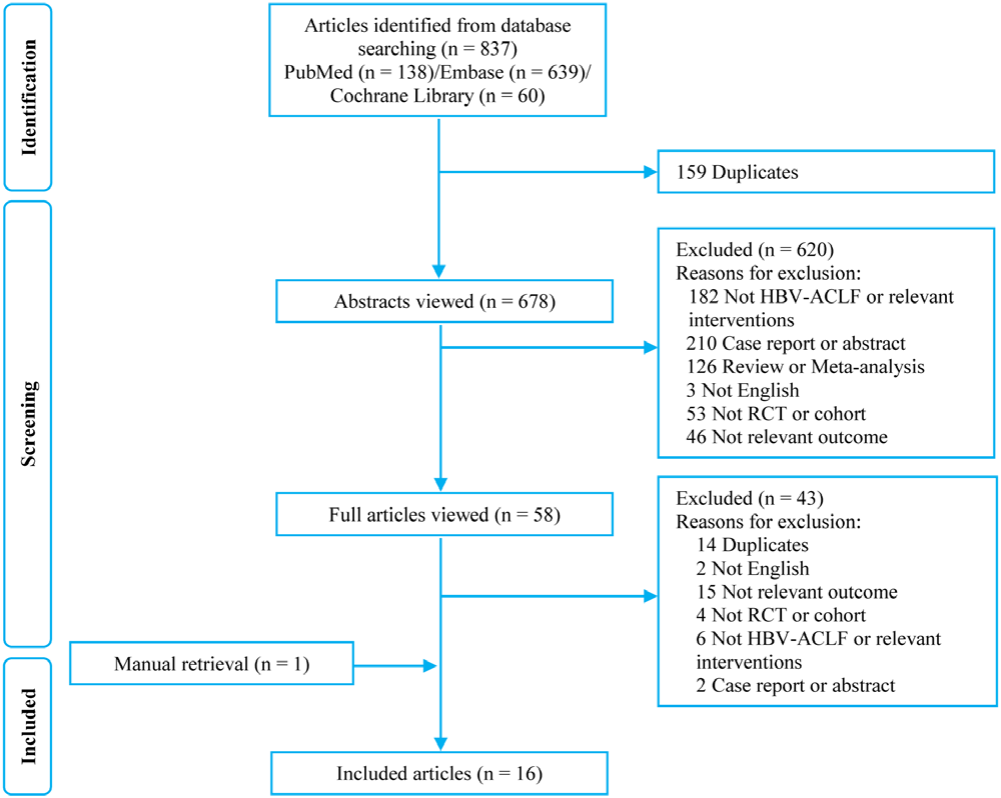
Process for identifying studies eligible for the meta-analysis. HBV-ACLF = hepatitis B virus related acute-on-chronic liver failure, RCT = randomized controlled trial.

A total of 837 studies were obtained based on the search methodology at the initial exploration. After removing the duplicate studies by screening the titles and abstracts, 58 studies with complete texts were potentially eligible. Ultimately, 16 studies were incorporated into the quantitative data synthesis.^[4,5,13–26]^ Figure 1 outlines the process of the systematic exploration and study selection. The characteristics of the included studies were summarized in Table 1. Besides, baseline clinical and experimental characteristics for each therapeutic method were shown in Table 2. All of these studies were conducted between 2008 and 2022, and a total of 2373 participants were included. Each of these studies had an intervention group and control group, which including PE versus NAs,^[5,15–17,19]^ GC versus NAs^,[20–22]^ G-CSF versus NAs,^[23,24]^ MSC versus NAs,^[25,26]^ MSC + PE versus PE,^[14]^ PE versus G-CSF versus MSC + PE^,[13]^ PE versus DPMAS + PE versus NAs^,[4]^ PE versus MSC versus PE + MSC versus NAs.^[18]^ The number of participants in all the studies ranged from 43 to 332, with the mean ages of patients with HBV-ACLF in all the studies ranging from 35 to 51.4 years.

**Table 1:** Characteristics of the included studies.

Year	Authors	Countries	Types of study	Sample size	Experimental group			Control group		
					Mean age	Sex (male)	Intervention	Mean age	Sex (male)	Intervention
2020	Zhu et al	China	RCT	60	43.95 ± 9.32	20 (100%)	PE	45.12 ± 7.14/40.10 ± 8.15	19 (95%)/20 (100%)	G-CSF/MSC + PE
2022	Wu et al	China	Prospective cohort	186	44.37 ± 11.71	56 (90.32%)	DPMAS + PE	48.23 ± 11.44/49.06 ± 13.72	56 (90.32%)/54 (87.10)	PE/NAs
2016	Li et al	China	Prospective cohort	45	51.10 ± 11.20	8 (72.7%)	MSC + PE	50.00 ± 10.90	26 (76.5%)	PE
2015	Wan et al	China	Retrospective cohort	158	51.40 ± 5.60	27 (71.1%)	PE	52.10 ± 6.60	101 (85%)	NAs
2019	Liu et al	China	Retrospective cohort	132	48.27 ± 11.93	63 (80.77%)	PE	50.61 ± 12.57	43 (79.63%)	NAs
2008	Yu et al	China	RCT	280	45.20 ± 10.20	112 (80%)	PE	46.40 ± 11.30	110 (78.6%)	NAs
2021	Chen et al	China	Prospective cohort	332	47.47 ± 11.40	145 (87.35%)	PE	47.30 ± 11.35	22 (8%)	NAs
2019	Xu et al	China	RCT	110	42.00 ± 6.55	20 (100%)	PE + MSC	40.87 ± 12.17/40.67 ± 9.89/44.97 ± 11.83	27 (90%)/29 (96.67%)/28 (93.3%)	PE/MSC/NAs
2014	Qin et al	China	RCT	234	44.13 ± 17.03	86 (82.69%)	PE	48.66 ± 18.55	94 (72.31%)	NAs
2022	Gao et al	China	Prospective cohort	280	35	161 (78.92%)	GC	38	58 (76.32%)	NAs
2013	Chen et al	China	Retrospective cohort	66	39.46 ± 11.92	33 (94.29%)	GC	36.94 ± 11.32	28 (90.32%)	NAs
2020	Jia et al	China	RCT	171	43.70 ± 12.70	74 (89.1%)	GC	46.60 ± 11.80	78 (88.6%)	NAs
2022	Tong et al	China	RCT	111	42.50 ± 10.20	44 (81.5%)	G-CSF	45.30 ± 10.60	47 (82.5%)	NAs
2013	Duan et al	China	RCT	55	43.5	21 (81.5%)	G-CSF	45.90	22 (78.6%)	NAs
2017	Lin et al	China	RCT	110	40.04 ± 9.9.4	51 (91.07%)	MSC	42.78 ± 8.40	53 (98.15%)	NAs
2012	Shi et al	China	RCT	43	40	20 (83.3%)	MSC	45	15 (78.9%)	NAs

DPMAS = double plasma molecular absorption system, GC = glucocorticoid; G-CSF = granulocyte colony-stimulating factor, MSC = mesenchymal stem cell, NA = nucleos(t)ide analogue, PE = plasma exchange, RCT = randomized controlled trial.

**Table 2:** The baseline clinical and experimental characteristics for each therapeutic method.

	Male, n (%)	Age (yr)	Albumin (g/L)	TBIL (µmol/L)	ALT (U/L)
PE					
Zhu et al	20 (100)	43.95 ± 9.32	29.70 ± 4.96	401.46 ± 121.67	136.95 ± 95.10
Wu et al	56 (90.32)	48.23 ± 11.44	30.57 ± 3.31	413.59 ± 174.23	370.80 (179.48, 634.33)
Li et al	26 (76.5)	50.0 ± 10.9	28.1 ± 6.2	336.6 ± 71.4	216.4 ± 153.5
Wan et al	27 (71.1)	51.4 ± 5.6	26.7 ± 1.43	19.8 ± 3.37	193.7 ± 33.4
Liu et al	63 (80.77)	48.27 ± 11.93	30.31 ± 4.89	308.55 (240.6, 387.7)	247 (88, 770)
Chen et al	145 (87.35)	47.47 ± 11.40	31.79 ± 5.70	437.48 + 162.20	188.00 (70.65, 607.50)
Xu et al	27 (90.00)	40.87 ± 12.17	35.44 ± 3.79	501.81 ± 135.53	234.57 ± 238.56
Qin et al	86 (82.69)	44.13 ± 17.03	33.31 ± 7.44	17.95 ± 8.25	501.5 ± 580.6
G-CSF					
Zhu et al	19 (95)	45.10 ± 7.14	31.35 ± 3.72	390.92 ± 118.66	136.15 ± 99.21
Tong et al	44 (81.5)	42.50 ± 10.20	29.0 (27.0–33.0)	324.4 (244.9–395.1)	111.0 (62.5–300.0)
Duan et al	21 (81.5)	43.5	29.11 ± 4.05	336 (181–519)	276 (197–801)
MSC					
Xu et al	29 (96.67)	40.67 ± 9.89	34.57 ± 4.24	455.78 ± 117.61	289.30 ± 594.25
Lin et al	51 (91.07)	40.04 ± 9.9.4	35.92 ± 4.34	495.24 ± 164.43	122.25 ± 91.88
Shi et al	20 (83.3)	40	24–44	124.7–631.3	17–1065
GC					
Gao et al	161 (78.92)	35	31.70 (29–34.78)	276.70 (184.58–415.03)	172.5 (91–397.5)
Chen et al	33 (94.29)	39.46 ± 11.92	34.37 ± 2.95	39.46 ± 11.92	1511.58 ± 535.92
Jia et al	74 (89.1)	43.70 ± 12.70	31.4 ± 3.6	335.3 ± 133.8	347.0 (131.7–685.9)
MSC + PE					
Zhu et al	20 (100)	40.10 ± 8.15	30.70 ± 2.99	358.63 ± 99.17	123.85 ± 74.93
Li et al	8 (72.7%)	51.1 ± 11.2	26.0 ± 4.7	297.8 ± 42.2	305.1 ± 164.9
Xu et al	20 (100.00)	42.00 ± 6.55	35.60 ± 4.70	542.86 ± 149.65	168.45 ± 149.75
DPMAS+PE					
Wu et al	56 (90.32)	44.37 ± 11.71	30.5 ± 3.61	384.11 ± 117	496.20 (208.83, 946.93)

ALT = alanine aminotransferase, DPMAS = double plasma molecular absorption system, GC = glucocorticoid; G-CSF = granulocyte colony-stimulating factor, MSC = mesenchymal stem cell, PE = plasma exchange.

### 3.2. Effects of different therapeutic methods on the 90-day survival rate of patients with HBV-ACLF

**Figure 2.**
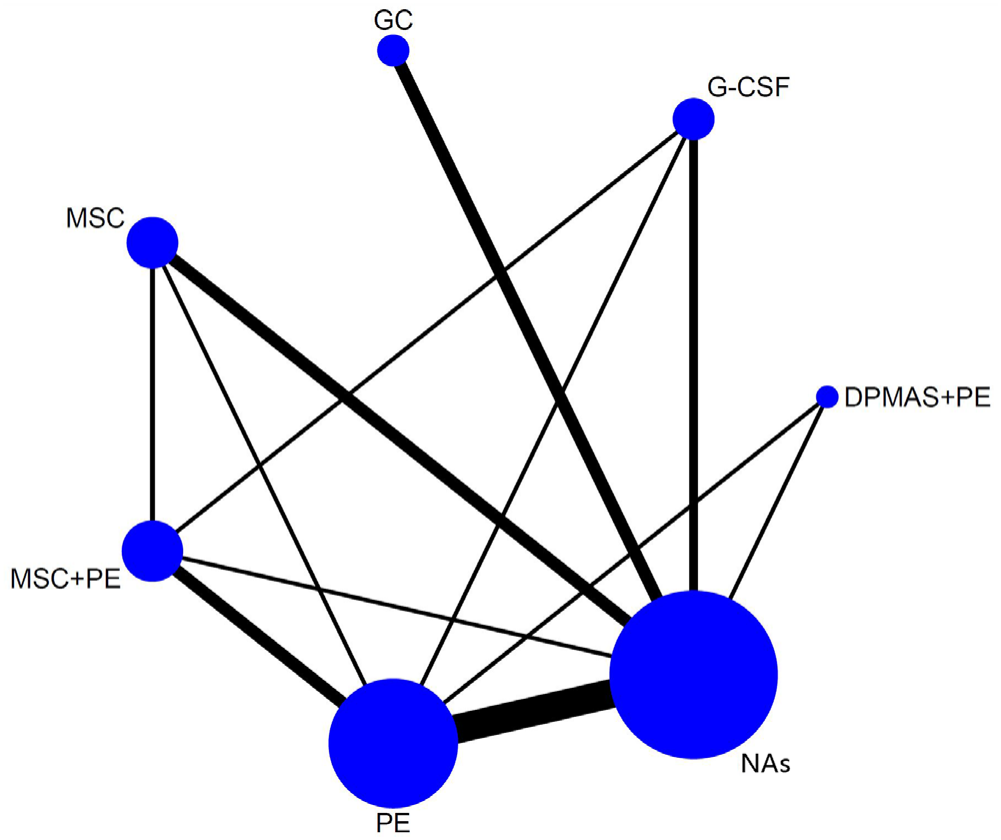
Network of eligible treatment comparisons for 90-day survival rate. DPMAS = double plasma molecular absorption system, GC = glucocorticoid; G-CSF = granulocyte colony-stimulating factor, MSC = mesenchymal stem cell, NA = nucleos(t)ide analogue, PE = plasma exchange.

**Figure 3.**
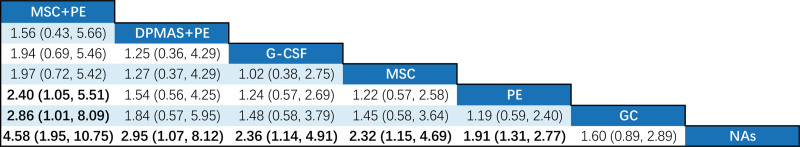
Comparison of the therapeutic effects of different treatment options in terms of the 90-day survival rate in patients with HBV-ACLF. The results were shown in boldface type when significant difference was observed between the 2 groups. DPMAS = double plasma molecular absorption system, GC = glucocorticoid; G-CSF = granulocyte colony-stimulating factor, HBV-ACLF = hepatitis B virus related acute-on-chronic liver failure, MSC = mesenchymal stem cell, NA = nucleos(t)ide analogue, PE = plasma exchange.

**Figure 4.**
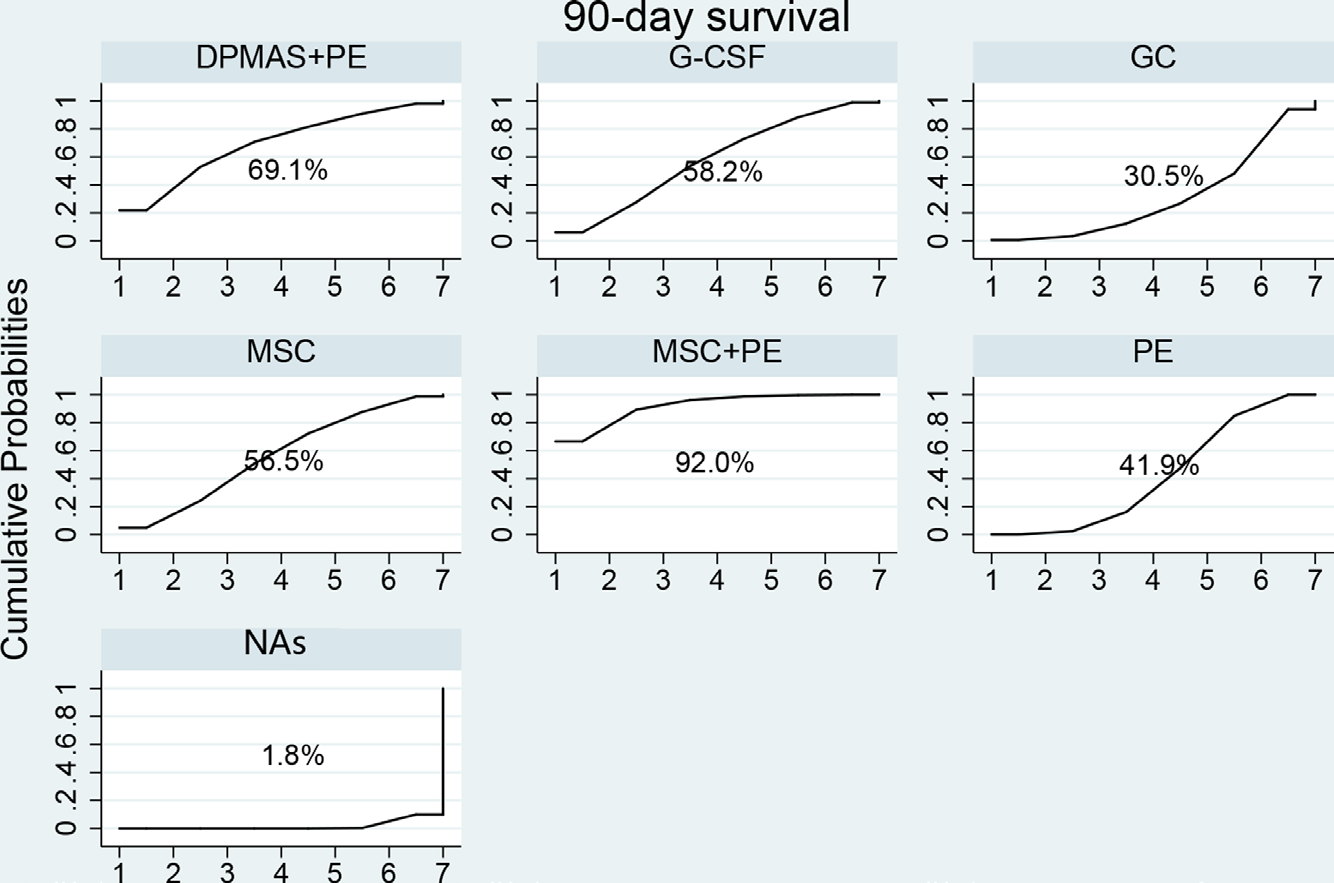
Ranking of treatment strategies based on probability of their effects on outcomes of 90-day survival rate according to the cumulative ranking area (SUCRA). Larger probability, stronger protective effects. DPMAS = double plasma molecular absorption system, GC = glucocorticoid; G-CSF = granulocyte colony-stimulating factor, MSC = mesenchymal stem cell, NA = nucleos(t)ide analogue, PE = plasma exchange, SUCRA = survival rate according to the cumulative ranking area.

Data concerning the 90-day LT-free survival rate of HBV-ACLF patients treated by different methods were available from all studies. These data were extracted and analyzed, the results were presented with the trial network plots (Fig. 2). The thickness of the line reflects the number of trials conducted to compare 2 treatment options, while the size of the node corresponds to the number of HBV-ACLF patients that were randomized to receive a particular treatment. Compared to NAs treatment, all of MSC + PE, DPMAS + PE, G-CSF, MSC and PE treatment possess the high odds of a 90-day survival rate (OR = 4.58, 95% CI: 1.95–10.75; OR = 2.95, 95% CI: 1.07–8.12; OR = 2.36, 95% CI: 1.14–4.91; OR = 2.32, 95% CI: 1.15–4.69; OR = 1.91, 95% CI: 1.42–2.77). Additionally, the odds of 90-day survival rate of MSC + PE treatment were superior to PE (OR = 2.40, 95% CI: 1.05–5.51) and GC (OR = 2.86, 95% CI: 1.01–8.09) treatment (Fig. 3). The effects of all the therapeutic methods on HBV-ACLF were ranked with SUCRA probabilities (Fig. 4), and the results indicated that MSC + PE had the greatest probabilities (SUCRA = 92%) of being the best treatment option for HBV-ACLF patients to improve the prognosis, followed by DPMAS + PE (SUCRA = 69.1%). For the monotherapy, G-CSF had the highest probabilities (SUCRA = 58.2%) of being the best treatment option for HBV-ACLF patients to improve the prognosis, followed by MSC (SUCRA = 56.5%), GC (SUCRA = 30.5%), and NAs (SUCRA = 1.8%).

### 3.3. Effects of different therapeutic methods on liver function of patients with HBV-ACLF

In this analysis, due to significant difference in treatment dosages and measurement time points among the studies, serum ALT and TBIL levels, along with MELD scores, were not subjected to pooled analysis. Liver function indicators were assessed in 13 studies, with detailed data presented in Table 3. Serum TBIL level changes were documented in 12 studies.^[4,5,13–15,17,18,21–23,25,26]^ Following treatments with PE, MSC, MSC + PE, and DPMAS + PE, a significant reduction in serum TBIL levels was observed in HBV-ACLF patients. As for the impact of GC treatment on serum TBIL levels, divergent outcomes were noted: Chen et al reported no significant changes,^[21]^ whereas Jia et al observed reductions on days 3 and 7 posttreatment.^[22]^ MELD scores were evaluated in 7 studies.^[14,17,18,21,24–26]^ Significant reductions in MELD scores were seen with PE, G-CSF, MSC, and MSC + PE treatments, unlike the GC treatment, which was reported by Chen et al to have no significant effect on MELD scores.^[21]^ Serum ALT levels were assessed in 9 studies.^[4,5,13–15,18,21,25,26]^ Significant decreases in serum ALT levels were reported in HBV-ACLF patients treated with MSC, MSC + PE, and DPMAS + PE. The effect of PE treatment on ALT levels was inconsistent, with 2 studies showing no improvement^[5,15]^ and 2 others indicating significant reductions.^[4,18]^

**Table 3:** Effects of different therapeutic methods on the liver function in included studies.

Author, publication year	Interventions	Dose	Time frame	Number of patients	Result
Zhu et al (2020)	MSC + PE	PMBC, 10^9^–10^10^/L, 60–90 mL, hepatic artery	All patients were followed at baseline, 4 d, 1, 2, 4, 12, and 24 wk	20	In the MSC + PE group, MELD score showed significant differences in the 4th week of treatment (*P* < .05). ALT showed not significantly difference between MSC + PE and PE groups
Wu et al (2023)	DPMAS + PE	–	Every 28–90 d	62	In the DPMAS + PE/PE group, the posttreatment levels of TBIL and ALT/AST were significantly lower than the pretreatment levels (*P* < .05). The rates of decrease in TBIL/AST in the DPMAS + PE group were significantly greater than those in the PE group (*P* < .05)
Li et al (2016)	MSC + PE	Two units of UC-MSCs (100 × 10^6^ cells suspended in 60 mL saline), hepatic artery	Patients were followed up every 2–4 weeks for 1–3 mo and every 3–6 mo for 4–24 mo	11	At 2 wk, patients in group PE had significantly lower ALT/AST s than that in PE, at 4 wk, patients in group MSC + PE showed significantly lower TBIL compared to PE (*P* < .05)
	PE	2–3 times/weekly		34	
Wan et al (2015)	PE	2–5 sessions	Followed up every 2–8 wk during therapy	38	There was no significant difference in ALT levels between the PE and NAs from week 2 onwards. TBIL in the PE group decreased significantly to 15 mg/dL by week 4
Yu et al (2008)	PE	Twice every week	Followed up for at least 3 mo	140	The levels of TBIL and MELD score in patients were significantly lower than before PE treatment (*P* < .05)
Chen et al (2021)	PE	–	On days 4, 7, 14, 21, and 28	166	Significant reductions in TBIL were observed only on day 7 in those treated with NAs + PE compared with those treated with NAs. ALT/AST levels in the NAs + PE group were lower in patients treated with NAs + PE on days 4, 7, 14, 21, and 28, and thereafter, the difference was not significant
Xu et al (2019)	PE	2 times a week, in a total of 3–5 times	At baseline and 30, 60, 90, 180, and 360 d	30	In the PE and PE + UC-MSC, TBIL and MELD score and AST/ALT significantly differed before and after each PE treatment. Among 3 groups, in mix-designed 2-way ANOVA analysis, the significant differences across time were found in TBIL, MELD score (*P* < .05)
	MSC	Allogeneic UC-MSC, once a week for 4 wk		30	
	MSC + PE	Combination of PE and MSC treatments		20	
Chen et al (2013)	GC	Group DMT underwent an injection of dexamethasone for the first 3 d (10 mg/d/person) intravenously	At baseline, 1, 2, 4, 8, and 12 wk, as well as the fourth day after enrollment	31	The results showed that no marked differences were found between the GC and NAs in the levels of ALT, TBIL and MELD score; compared to baseline, GC decrease ALT at the end of 3-d steroid therapy (*P* < .05)
Jia et al (2020)	GC	1.5 mg/kg/d, days 1–3; thereafter 1 mg/kg/d, days 4–5; and followed by 0.5 mg/kg/d, days 6–7	At the baseline (0 d), 3 d, 7 d, 10, 14, 30d, and then monthly until the 6th month.	83	Compared to control group, serum bilirubin was lower on day 3 and day 7
Tong et al (2022)	G-CSF	G-CSF was injected subcutaneously at a dose of 5 mg/kg every day for 6 ds and then every other day until day 18	At least 180 d after treatment commencement	56	Serum bilirubin in the G-CSF group were higher than those in the control group, but the differences were not statistically significant
Duan et al (2013)	G-CSF	G-CSF subcutaneously at the dosage of 5 μg/kg/d for 6 consecutive days	All patients had daily follow-ups and physical examinations in the first month, and then at least weekly for the next 2 mouth	27	The MELD scores demonstrated an early decrease in the G-CSF group on the day 7, 15, 30 (*P* < .05)
Lin et al (2017)	MSC	The MSC group received infusions of 1.0–10 × 10^5^ cells/kg allogeneic BM-MSCs via peripheral veins once a week for 4 wk	Observation and follow-up data were recorded from immediately before the first infusion and at 1, 2, 3, 4, 8, 12 and 24 wk afterwards	56	During the first 4/24 wk, the improvement of TBIL and MELD score in the MSC group was significantly greater compared with that in the NAs group. The MELD scores in the MSC group had decreased more dramatically than NAs group at weeks 1 and 2. The levels of ALT in the MSC group had improved more significantly than in the NAs group at week 1
Shi et al (2012)	MSC	Approximately 0.5 × 10^6^ UC-MSCs per kilogram of body weight, infused intravenously, 3 times at 4-wk intervals	The following liver function tests were performed on weeks 1, 2, 4, 8, 12, 24, 36, and 48	24	TBIL decreased significantly as compared with baseline after UC-MSC transfusion. The MELD scores were decreased more in the UC-MSC-treated patients than the control group at weeks 4, 8, and 12. After UC-MSC treatment, the ALT levels decreased significantly compared with the baseline throughout the 48 wk of follow-up

ALT = alanine aminotransferase, DPMAS = double plasma molecular absorption system, GC = glucocorticoid; G-CSF = granulocyte colony-stimulating factor, MELD = model of end-stage liver disease, MSC = mesenchymal stem cell, NA = nucleos(t)ide analogue, PE = plasma exchange, TBIL = total bilirubin, UC-MSC = umbilical cord-derived mesenchymal stem cell.

### 3.4. Safety of different therapeutic methods in patients with HBV-ACLF

The safety profiles of different therapeutic interventions in HBV-ACLF patients were reported across several studies. Adverse effects of PE were noted in 2 articles,^[4,14]^ with incidence rates of 18.13% and 27.3%, respectively. The reported side effects included rash and hypotension, which were alleviated with antiallergic therapy. Regarding GC treatment, Jia et al reported an incidence rate of infections of 41.0% in the GC group, compared to 31.8% in the control group.^[22]^ Similarly, Gao et al described a 31.37% incidence rate of infections in the GC treatment group, which exceeded that in the control group (22.37%).^[20]^ However, neither study demonstrated statistical significance in these findings. Concerning G-CSF treatment,^[23,24]^ the prevalent side effects included fever, headache, and nausea. No severe adverse effects were reported during the G-CSF treatment period, and the occurrence of complications did not significantly differ from that of the control group. In the context of MSC therapy, 1 study highlighted self-limiting fever as a noteworthy side effect.^[25]^

### 3.5. Estimating the consistency of the treatment effects in the included studies

**Figure 5.**
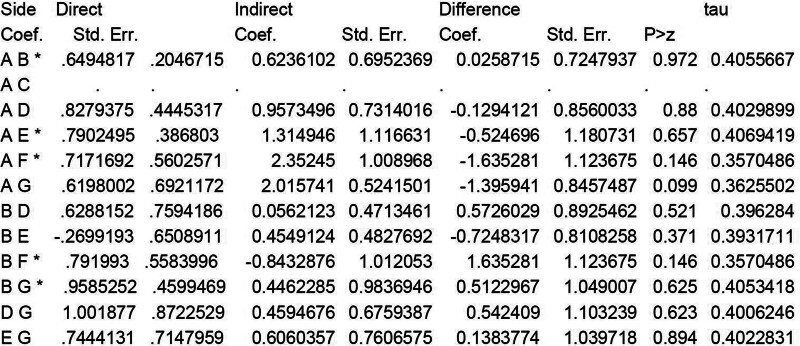
Estimation of the consistency of the treatment effects among the included studies by node splitting.

To investigate the consistency of the treatment effects among the included studies, the loop consistency was evaluated utilizing node splitting to identify the similarities between direct and indirect treatment effects. Figure 5 presents the direct and indirect estimates obtained through node splitting. The treatment networks did not reveal any loops that were significantly inconsistent. Furthermore, the data were double-checked thoroughly and no significant effect modifier that varied across the comparisons was identified.

### 3.6. Estimating the quality and publication bias of the included studies

**Figure 6.**
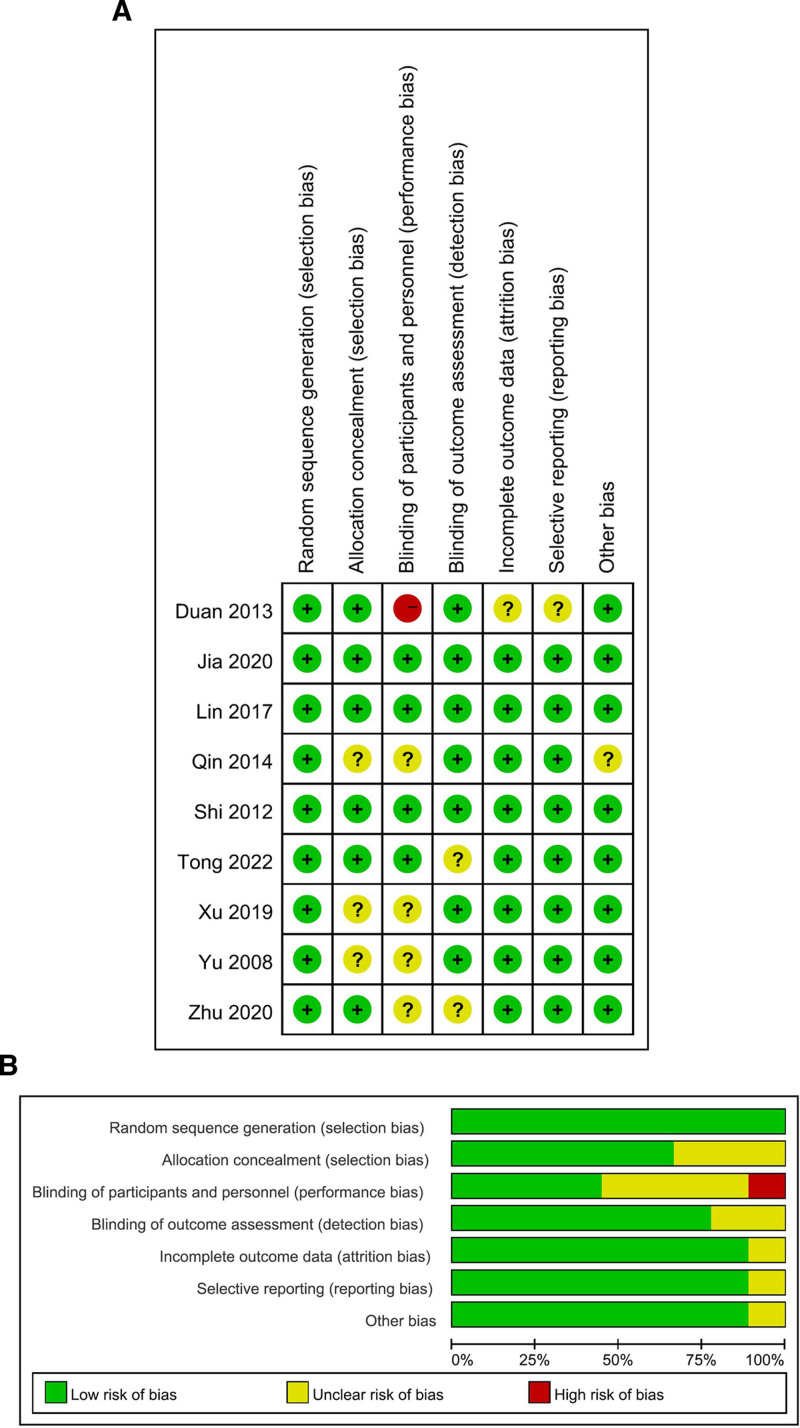
Quality assessment of the included RCTs. RCT = randomized controlled trial.

**Figure 7.**
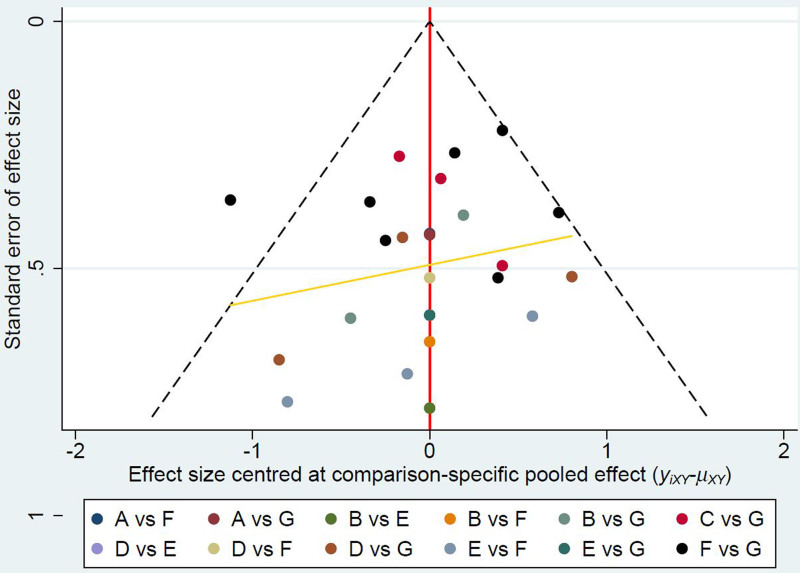
Publication bias assessment by the funnel plot.

Quality assessment was conducted to investigate the quality of included studies. With regard to the RCTs, there were 3 high-quality studies, 5 moderate-quality studies and 1 low-quality study. The main sources of bias were the absence of proper random sequence generation, concealment of allocation, and description of blinding. With regard to the cohort studies, the mean Newcastle-Ottawa scale score was 6.5, which represents a relatively high quality of these cohort studies (Fig. 6 and Table 4). The publication bias was assessed by the funnel plot, and the results indicated that no obvious publication bias was observed in the included studies (Fig. 7).

**Table 4:** Quality assessment of the included cohort studies.

Author	Representativeness of the exposed cohort	Selection of the nonexposed cohort	Ascertainment of exposure	Demonstration that outcome of interest was not present at start of study	Comparability of cohorts based on the design or analysis	Assessment of outcome	Was follow-up long enough for outcomes to occur	Adequacy of follow-up of cohorts	Total scores
Wu et al (2022)	*	*	*	*	**	*	–	*	7
Li et al (2016)	*	*	–	*	**	*	–	–	6
Wan et al (2015)	*	*	*	*	**	–	–	–	6
Liu et al (2019)	*	*	-	*	**	–	*	–	6
Chen et al (2021)	–	*	*	–	**	*	*	–	6
Gao et al (2022)	*	*	*	–	**	*	*	*	8
Chen et al (2013)	*	*	*	*	*	*	–	*	7

* *P* < .05.

** *P* < .01.

## 4. Discussion

HBV-ACLF causes a high mortality worldwide, and several therapeutic methods have been developed for HBV-ACLF, such as NAs, PE, GC, DPMAS, MSC, G-CSF.^[8–11]^ Although all of these therapeutic methods are effective for HBV-ACLF, comparison of their curative effects remains unclear. This network meta-analysis was carried out to summarize the therapeutic effect of each method in HBV-ACLF patients and to evaluate the transplant-free survival at 3 months, as well as the safety of each therapeutic method. After searching the database and comprehensively screening all the relevant literature, 16 studies comprising 2373 patients, were included in this meta-analysis. The results of this study indicated that combined therapy of MSC + PE is the most effective therapeutic method for HBV-ACLF patients in terms of 90-day survival rate. For single-drug regimens, G-CSF possesses the highest therapeutic potential for HBV-ACLF, followed by MSC and GC.

Several pooled meta-analyses had already examined the efficacy and safety of the therapeutic methods which involved in this study in HBV-ACLF patients. Chen et al conducted a pooled meta-analysis to investigate the therapeutic effect of MSC on patients with HBV-ACLF, and the results indicated that MSC treatment could significantly reduce the mortality rate without increased risk of severe complications.^[27]^ At present, umbilical cord-derived mesenchymal stem cells (UC-MSCs) are commonly used in stem cell therapy with multiple advantages, including easy isolation and harvesting, low tumorigenicity, low immunogenicity, and no ethical concerns.^[28]^ Cheng et al have shown that UC-MSCs transplantation could increase the survival rate by improving liver function without severe adverse events, and possess the superior therapeutic choose for patients with HBV-ACLF.^[29]^ Additionally, Zhang et al found that the therapeutic effect of UC-MSCs vary depending on the patient’s age, highlighting the importance of age assessment before UC-MSCs clinical use.^[30]^ Although the specific mechanism of MSC in the treatment of HBV-ACLF remain unclear, the differentiated potential of MSC into hepatocyte-like cells was initially established by the discovery of hepatocyte-like cells containing Y-chromosomes in the liver of a female recipient who had undergone allogenic blood MSC transplantation from male donors.^[31]^ Additionally, MSC could exert immunomodulatory effects by cell-to-cell contact paracrine activity with immune cells to suppress the early excessive immune response in HBV-ACLF patients.^[32]^ Furthermore, MSC could impede the activation of stellate cells by releasing IL-10 and TNF-α, ultimately resulting in a reduction in liver fibrosis, which was particularly important for the post-recovery of HBV-ACLF patients.^[33,34]^ In this study, the results showed that after MSC treatment, there was a significant improvement on liver function of patients with HBV-ACLF, as measured by serum TBIL and ALT levels, and MELD scores, and lower infection risk, which led to an improved prognosis for HBV-ALCF patients. Furthermore, the results indicated that MSC + PE is the optimal treatment option for improving the 90-day survival rate of HBV-ACLF patients, and the 90-day survival rate of HBV-ACLF patients treated with MSC + PE was superior to that patients treated with PE alone. To sum up, MSC + PE therapy could be regarded as the primary therapeutic method to constrain the overwhelming fatality rate in patients with HBV-ACLF.

Norberto et al demonstrated that treatment with G-CSF could significantly reduce the overall mortality of HBV-ACLF patients, while increase peripheral neutrophil/leukocyte counts, as well as the peripheral and intrahepatic CD34 + cell counts.^[35]^ Huang et al reported in a small-scale meta-analysis that treatment with G-CSF could effectively enhance the survival rate of patients with ACLF during the first 3 months.^[36]^ Duan et al also founded that the peripheral neutrophil and CD34 + cell counts in G-CSF treatment group were increased on day 3 from the onset of therapy, continued to rise on day 7, and remained elevated on day 15.^[24]^ Interestingly, the results of this study showed that G-CSF treatment was the most effective monotherapy to improve the 90-day prognosis of HBV-ACLF patients compared to MSC, PE and GC, which suggested that G-CSF should be the recommended monotherapy for HBV-ACLF.

The previous guidance (APASL 2019) recommended that PE could be regarded as a promising and effective bridging therapy in patients with ACLF to liver transplant or spontaneous regeneration.^[1]^ Multiple clinical trials have also confirmed the effectiveness of PE in improving the prognosis of ACLF. Tan et al found that PE treatment could improve the survival rate at 30- and 90-days in non-transplanted ACLF patients.^[37]^ Additionally, a meta-analysis conducted by Ocskay et al showed that PE seems to be the best liver support therapy currently in ACLF regarding 3-month overall survival.^[38]^ Regarding the differences between PE and DPMAS, a prospective study had found that although there was no significant difference in 90-day survival rate for PE and DPMAS, the serum levels of albumin, TBIL, and C-reactive protein in DPMAS group were superior in the PE group.^[39]^ The combination of DPMAS and PE can effectively eliminate toxins and inflammatory factors, and supply nutrients at the same time, thus making up for their respective shortcomings. Bai et al also found that compared with PE treatment, DPMAS + PE can improve the survival rate, serum ALT and hemoglobin levels.^[40]^ In this meta-analysis, the results also support that DPMAS + PE treatment can alleviate the liver function of HBV-ACLF patients by decreasing the serum levels of TBIL and ALT, and the MELD scores.

There are several limitations in this study. Firstly, the sample sizes were relatively small in several included studies. Secondly, the number of RCTs in included studies was relatively small. Thirdly, all enrolled patients were of Chinese ethnicity, which may generate bias for the results. Further studies should be conducted in other countries and ethnicities. Furthermore, the optimal treatment scheme and additional RCTs consisting of a large number of participants are necessary to validate the effects of different therapeutic methods on the treatment of HBV-ACLF patients. Although the conclusions of this study support the superiority of MSC + PE over other therapeutic approaches in improving the prognosis as indicated by the 90-day survival rate, the safety of MSC + PE in practical clinical applications still merits significant attention. When different patients are treated with MSC + PE, the source and preparation process of MSC, the dose and route of administration of MSC need to be closely reviewed, and the influence of individual differences on the efficacy of MSC therapy should be comprehensively considered. In addition, the long-term safety of MSC still warrants further investigation.

## 5. Conclusions

In summary, this meta-analysis investigated the effects of different therapeutic methods on the treatment of HBV-ACLF, and the results showed that MSC + PE was superior to other therapeutic methods in improving the prognosis as indicated by the 90-day survival rate, and G-CSF was found to be the most effective monotherapy in improving the 90-day prognosis of patients with HBV-ACLF. In addition, MSC + PE could improve the liver function by decreasing the serum levels of TBIL and ALT, and the MELD scores. However, it is important to interpret these findings cautiously due to the presence of certain limitations in this study. Further high-quality randomized clinical trials should be conducted to investigate the treatment timing, dosing, and treatment intervals of different therapeutic methods to achieve the optimal therapeutic effect for patients with HBV-ACLF.

## Acknowledgments

We thank Xuefeng Ma for the assistance in this study.

## Author contributions

**Conceptualization:** Shousheng Liu, Yongning Xin.

**Data curation:** Yin Hua, Yuqin He.

**Formal analysis:** Yin Hua, Huaqiang Liu, Yuqin He.

**Methodology:** Shousheng Liu.

**Resources:** Yin Hua, Huaqiang Liu, Yuqin He.

**Supervision:** Yongning Xin.

**Writing – original draft:** Yin Hua.

**Writing – review & editing:** Shousheng Liu, Yongning Xin.
